# Asthma Heredity, Cord Blood IgE and Asthma-Related Symptoms and Medication in Adulthood: A Long-Term Follow-Up in a Swedish Birth Cohort

**DOI:** 10.1371/journal.pone.0066777

**Published:** 2013-06-21

**Authors:** Hartmut Vogt, Lennart Bråbäck, Olof Zetterström, Katalin Zara, Karin Fälth-Magnusson, Lennart Nilsson

**Affiliations:** 1 Division of Pediatrics, Department of Clinical and Experimental Medicine, Faculty of Health Sciences, Linköping University, Pediatric and Adolescent Clinic, County Council of Östergötland, Linköping, Sweden; 2 Department of Research and Development, Västernorrland County Council, Sundsvall, Sweden; 3 Occupational and Environmental Medicine, Department of Public Health and Clinical Medicine, Umeå University, Umeå, Sweden; 4 Allergy Centre, Department of Clinical and Experimental Medicine, Faculty of Health Sciences, Linköping University, Linköping, Sweden; Baylor College of Medicine, United States of America

## Abstract

Cord blood IgE has previously been studied as a possible predictor of asthma and allergic diseases. Results from different studies have been contradictory, and most have focused on high-risk infants and early infancy. Few studies have followed their study population into adulthood. This study assessed whether cord blood IgE levels and a family history of asthma were associated with, and could predict, asthma medication and allergy-related respiratory symptoms in adults. A follow-up was carried out in a Swedish birth cohort comprising 1,701 consecutively born children. In all, 1,661 individuals could be linked to the Swedish Prescribed Drug Register and the Medical Birth Register, and 1,227 responded to a postal questionnaire. Cord blood IgE and family history of asthma were correlated with reported respiratory symptoms and dispensed asthma medication at 32–34 years. Elevated cord blood IgE was associated with a two- to threefold increased risk of pollen-induced respiratory symptoms and dispensed anti-inflammatory asthma medication. Similarly, a family history of asthma was associated with an increased risk of pollen-induced respiratory symptoms and anti-inflammatory medication. However, only 8% of the individuals with elevated cord blood IgE or a family history of asthma in infancy could be linked to current dispensation of anti-inflammatory asthma medication at follow-up. In all, 49 out of 60 individuals with dispensed anti-inflammatory asthma medication at 32–34 years of age had not been reported having asthma at previous check-ups of the cohort during childhood. Among those, only 5% with elevated cord blood IgE and 6% with a family history of asthma in infancy could be linked to current dispensation of anti-inflammatory asthma medication as adults. Elevated cord blood IgE and a positive family history of asthma were associated with reported respiratory symptoms and dispensed asthma medication in adulthood, but their predictive power was poor in this long-time follow-up.

## Introduction

Cord blood IgE (CB-IgE) as a possible predictor of asthma and allergy has been evaluated in a number of studies in recent years. These investigations have shown conflicting results [1], [2], [3], [4], [5]. Moreover, very few studies have followed the children into adulthood and their conclusions have been contradictory [3], [5].

This study is part of a follow-up of one of the oldest and largest asthma and allergy birth cohorts. It was initially started in the mid-1970s by our colleagues Kjellman and Croner [6]. The birth cohort was investigated for the development and prediction of asthma and atopic disease up to the age of 12–14 years [7], [8], [9]. CB-IgE concentration discriminated better than family history between atopic and non-atopic subjects at 7 years, but combined information was considered useful [9]. Elevated CB-IgE was associated with a fivefold increased risk of asthma at 11 years, but sensitivity was only 26%. Neither CB-IgE nor a family history of atopic disease was found to be strong enough as a single screening method for atopic disease, including asthma, at 11 years [7]. In an English investigation raised cord serum IgE was found to be associated with asthma at 10 years of age, although the same association was lacking at an earlier follow-up at 4 years of age, perhaps due to the gradual development of asthma in this aging cohort as suggested by the authors [4]. Furthermore, a study from Canada also found an association between high CB-IgE and asthma symptoms at a later stage but not in a previous investigation of the same cohort [10]. Even if the prediction of asthma based on IgE levels at birth has not been of convincing value in later childhood or adulthood, it might still be a valuable indicator in certain high-risk cohorts.

The aim of our study was to assess whether elevated CB-IgE levels and a family history of asthma in early childhood were associated with, and could predict, allergy-related respiratory symptoms and dispensation of asthma medication at 32–34 years of age. We investigated all cases of adults with presumable asthma and specifically the cases that seemed to be diagnosed after the age of 11 years.

## Materials and Methods

### Ethics Statement

Study participants provided written informed consent by answering the postal questionnaires. The linkage of data to the National registers was approved and performed by the National Board of Health and Welfare and did not require any verbal or written consent as data were analyzed anonymously. The Regional Ethical Review Board in Linköping, Linköping University, approved all procedures and study protocols, and all procedures were in accordance with the Declaration of Helsinki.

### Study Population

The study is based on a follow-up of a Swedish asthma and allergy birth cohort containing all infants consecutively born from December 1974 to December 1975 at Linköping county hospital. Of 1,884 infants born during that period, 1,701 were able to be enrolled in the original study. Development of asthma and allergic disease in relation to both CB-IgE and family history, separately and in combination, was investigated at different time points [6], [7], [8], [9]. At the age of 6–7 and 10–11 years of age asthma and allergic disease status was evaluated by parental questionnaire, telephone check-ups and the review of available medical records. Obvious asthma was defined as recurrent wheezing, cough and breathlessness on at least three occasions when the child suffered from a cold or at least once after specific allergen exposure [7], [9].

In 2007 we performed a questionnaire-based follow-up of the original study population to investigate the current asthmatic and allergic status of these now adult individuals. Almost all former study participants could be identified by their personal identification number (PIN), a unique 10-digit number that all Swedish residents are assigned at birth. Forty-five individuals had to be excluded as either no valid address could be located or their PIN turned out to be incorrect. A total of 1,238 (72.8%) answered the postal questionnaire. An additional 11 individuals had to be excluded for various reasons, leaving 1,227 (72.1%).

**Figure 1:**
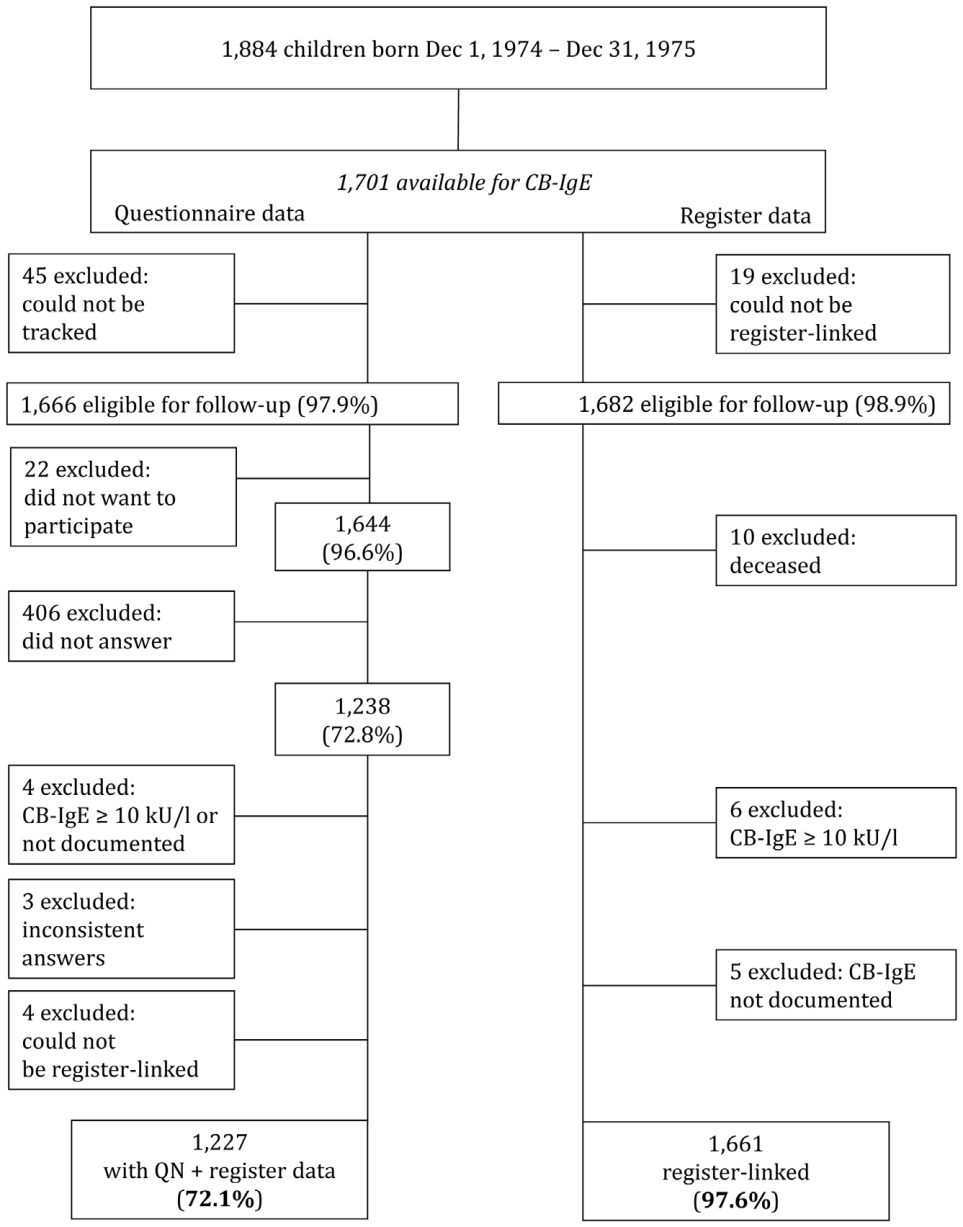
Flow chart of the study population with respect to questionnaire data and register data. CB-IgE = Cord blood Immunoglobulin E; PIN = Personal identification number; QN = Questionnaire.

In a second phase the same study population was linked to the Swedish Prescribed Drug Register and the Swedish Medical Birth Register. The registers linked to 1,661 (97.6%) individuals (Figure 1).

### Questionnaire Data

The questionnaire included 31 questions regarding respiratory symptoms as well as nose-eye symptoms, skin symptoms, current smoking status, occupation, diet, physical habits and residential status (“*Having lived on a farm”)* during the first 5 years of life. A positive answer to the question *“Do you become breathless, start to wheeze or cough due to contact with pollen from trees or grass?”* or *“… due to contact with furred animals?”* was used as a marker of respiratory symptoms due to pollen or furred pets.

In all, 32/1,227 chose not to answer the question for *pollen-induced* respiratory symptoms, and 42/1,227 did not answer the question for *pet-induced* respiratory symptoms. As these individuals provided valid responses to most of the other questions in the questionnaire, they were included in the analyses when possible.

*Season of birth* was categorized as winter (December – February), spring (March – May), summer (June – August) and fall (September – November) based on the date of birth.

Information about a positive *family history of asthma* was based on questionnaire data provided by the parents and collected when the children were originally evaluated at 18 months of age and implied that at least one of the parents or an older sibling was reported to have had asthma at the time point of evaluation of the child. Information about number and age of siblings was not documented.

### Register Data

We also used data from the Swedish Prescribed Drug Register about the study population’s dispensation of prescribed anti-asthmatic medicines during 2006–2008 as a proxy for asthma. This register contains data on all dispensed prescriptions for the entire Swedish population since July 1, 2005. Dispensed prescriptions are linked to the PIN. The register is owned and maintained by the Swedish National Board of Health and Welfare. Drug data are recorded according to their corresponding Anatomical Therapeutical Chemical (ATC) code [11].

*Any asthma medication* was defined as at least two dispensed prescriptions of either a selective β_2_-agonist (R03AC), an inhaled corticosteroid (R03BA) or a combination of a β_2_-agonist and another drug for obstructive airway disease (R03AK04 through R03AK07) or a leukotriene antagonist (R03DC03) during 2006–2008. *Anti-inflammatory treatment* included two or more dispensed prescriptions of an inhaled corticosteroid (including combinations of corticosteroids with other drugs; R03AK04 through R03AK07) and/or leukotriene antagonist during 2006–2008.

Information about such potential confounding factors as maternal age, gestational age, parity, small for gestational age (SGA), large for gestational age (LGA) and caesarean section was obtained from the Swedish Medical Birth Register. The national register was established in 1973 and includes prenatal data as well as data from the neonatal period on practically every child born in Sweden reported by members of the health care system. Register data on prenatal growth (SGA, LGA) are based on intrauterine growth curves and were missing for 26 of the 1,661 individuals. Data for gestational age were missing for 5 individuals and data for mother’s country of birth for 1 individual.

### Analysis of CB-IgE

Initially, serum from cord blood was separated and frozen to −20°C within 24 hours and stored pending analysis. Total IgE was measured, in duplicate, with Phadebas IgE PRIST (Pharmacia Diagnostics AB; Uppsala, Sweden). The test was calibrated for a detection limit of 0.9 kU/l [6].

Our data analyses were based on stored CB-IgE values. According to the detection limit, CB-IgE was dichotomized into <0.9 kU/l (undetectable) and ≥0.9 kU/l (elevated).

As the original blood samples were no longer available for analysis in 2007, contamination with maternal blood could not be tested. Therefore, to account for a probable fetomaternal contamination, four individuals with CB-IgE levels of 10.0 kU/l or above were excluded from the analysis as other investigators have proposed [10].

### Statistical Analysis

Statistical analyses were performed using IBM SPSS Statistics for Windows Release 19.0.0.

Chi-square was used for bivariate comparisons between CB-IgE level and different respiratory symptoms and anti-asthma medication, and to compare basic characteristics of the study population with respect to the two different outcomes measured. Fisher’s Exact Test was used in case there were cells with expected counts of less than five. Sensitivity, specificity, positive predictive value and positive likelihood ratio were calculated following standard statistical measures [12].

The flow of the study population with respect to asthma diagnosis at 6–7 years of age, at 10–11 years of age and anti-inflammatory asthma medication at 32–34 years of age was evaluated using common contingency tables.

Multivariate logistic regression analyses were performed to control for potential covariates such as gender, family history of asthma, having lived on a farm during the first five years of life, season of birth and various perinatal parameters, as mentioned above.

As a level of statistical significance, p was set at <0.05.

## Results

There were no significant differences in basic characteristics between the individuals that had answered the postal questionnaires (n = 1,227) and those who could be linked to the drug register (n = 1,661) in relation to CB-IgE values (Table 1).

**Table 1:** Basic characteristics of the study population with respect to low (<0.9 kU/l) and high (≥0.9 kU/l) CB-IgE and available data at follow-up (questionnaire+register data versus register data alone).

		CB-IgE		CB-IgE		
		QN+register data (n = 1,227)		Register data (n = 1,661)		
		<0.9 kU/l	≥0.9 kU/l	<0.9 kU/l	≥0.9 kU/l	
		N = 1,074	N = 153	N = 1,456	N = 205	p value
		%	%	%	%	
Sex	Female	55.8	44.4	51.0	39.5	0.795
Season of birth	Spring	29.8	28.1	29.7	28.3	0.999
	Summer	24.4	24.8	23.6	23.9	0.999
	Fall	19.6	19.6	19.2	22.0	0.707
	Winter	26.2	27.5	27.5	25.7	0.581
History of asthma	Family	5.6	9.2	6.0	9.8	0.999
	Mother	2.0	3.9	2.3	5.4	0.789
	Father	2.2	2.6	2.4	2.0	0.711
Mother’s age^1^	≤26 years	52.4	58.2	53.3	58.0	0.821
	>26 years	47.6	41.8	46.7	42.0	0.999
Gestational age^1,2^	Week 31–37	8.5	13.1	8.4	13.2	0.999
	Week 38–42	80.6	75.2	79.5	76.0	0.999
	Week 42+	10.9	11.8	12.1	10.8	0.608
Prenatal growth^3^	SGA	3.8	6.8	4.0	5.6	0.630
	LGA	2.2	1.4	1.9	1.5	0.999
Firstborn	Yes	41.8	42.5	41.5	41.5	0.872
Caesarean section	Yes	4.4	3.3	4.9	2.4	0.526
Mother’s country of	Scandinavia	99.3	98.0	99.2	98.0	0.954
birth^4^	Others	0.7	2.0	0.8	2.0	0.999

CB-IgE = Cord blood immunoglobulin E; QN = questionnaire; SGA = Small for Gestational Age; LGA = Large for Gestational Age;

1 entered as continuous variable in the regression model, dichotomized here for descriptive reasons;

2 n = 1,225/1,656;

3 n = 1,209/1,635;

4 n = 1,226/1,660.

Significance levels are displayed for the association between the different ratios of low versus high CB-IgE between the groups.

Of the 1,227 study members who answered to the postal questionnaire, 153 individuals (12.5%) had detectable levels of CB-IgE at birth. Among those linked to the drug register (n = 1,661), 205 (12.3%) had detectable levels of CB-IgE. CB-IgE values ranged from detection level 0.9 to 9.7 kU/l (Mean 1.76, median 0.90). Elevated CB-IgE-levels were significantly more common in males than in females in both groups (n = 1,227 and n = 1,661 with p = 0.009 and p = 001 respectively), and there was a significant difference in the register-linked group (n = 1,661) between the individuals with low CB-IgE compared to those with elevated CB-IgE with respect to a family history of asthma (p = 0.039) and a history of asthma in the mother (p = 0.010) (Table 1).

Of the 1,227 children included in the questionnaire follow-up, 74 individuals (6.0%) had a documented positive family history of asthma at 18 months of age. The corresponding number for the individuals linked to the drug register (n = 1,661) was 107 (6.4%). The exact distribution of asthma heredity (asthmatic mother and/or father and/or sibling) is displayed in Table S1.

The prevalence rates of self-reported respiratory symptoms induced by pollen and pets were 15.0% (179/1,195) and 9.7% (115/1,185), respectively. Of all individuals with pollen-induced respiratory symptoms, 20.1% (36/179) had an elevated level of CB-IgE, and 11.2% (20/179) had a family history of asthma during infancy. Corresponding rates among individuals with pet-induced symptoms were 20.9% (24/115) and 13.9% (16/115), respectively.

Among individuals linked to the drug register, rates of *any asthma medication* and any *anti-inflammatory* drugs with at least *two* purchases during 2006–2008 were 6.6% (110/1,661) and 3.6% (60/1,661), respectively. Of all individuals with any asthma medication, 20.9% (23/110) had an elevated level of CB-IgE, and 14.5% (16/110) had a family history of asthma during infancy. Corresponding rates among individuals with any anti-inflammatory medication were 26.7% (16/60) and 15.0% (9/60), respectively.

The prevalence rates of asthma, respiratory symptoms and asthma medication at the three different time points of follow-up with respect to gender are displayed in Table 2.

**Table 2:** Asthma prevalence (6–7, 10–11 years) and respiratory symptoms and asthma medication (32–34 years) with respect to gender.

	Males		Females		Total
	n	%	n	%	N
6–7 yrs	27	56.2	21	43.8	48
10–11 yrs	27	56.2	21	43.8	48
32–34 yrs					
Respiratory symptoms due to pollen^1^	79	44.1	100	55.9	179
Respiratory symptoms due to furred pets^2^	54	47.0	61	53.0	115
Any asthma medication	56	50.9	54	49.1	110
Anti-inflammatory treatment (all cases)	31	51.7	29	48.3	60
Anti-inflammatory treatment (only new cases)^3^	25	51.0	24	49.0	49

1 data available for 1195 individuals;

2 data available for 1185 individuals,

3 cases with no asthma neither at 6–7 nor at 10–11 years.

Reported respiratory symptoms and dispensation of asthma medication corresponded only partly. In all, 65.3% of those with at least two dispensed prescriptions of *any asthma medication* and 71.4% of those who purchased at least two prescriptions of *anti-inflammatory* drugs had reported respiratory symptoms due to pollen. The corresponding rates for respiratory symptoms due to contact with pets were 52.1% and 56.1%, respectively. Among those with respiratory symptoms due to pollen, only 27.4% had purchased at least two prescriptions of *any asthma medication* and 16.8% at least two purchases of an *anti-inflammatory* drug. For those with reported respiratory symptoms due to contact with furred pets, the corresponding rates were 33.0% and 20.0%, respectively.

Respiratory symptoms and asthma medication use were twice as common in those with high levels as among those with low levels of CB-IgE. Similar differences were found when comparing respiratory symptoms and asthma medication in relation to family history (Table 3).

**Table 3:** Prevalence of respiratory symptoms (2007) and dispensed asthma medication (2006–2008) at 32–34 years of age in relation to levels of CB-IgE and family history of asthma.

	CB-IgE					Family history of asthma				
	<0.9 kU/l		≥0.9 kU/l			negative		positive		
	n	%	n	%	p value		%		%	p value
Respiratory symptoms due to pollen^1^	143/1,046	13.7	36/149	24.2	0.001	159/1,124	14.1	20/71	28.2	0.003
Respiratory symptoms due to furred pets^2^	91/1,038	8.8	24/147	16.3	0.005	99/1,115	8.9	16/70	22.9	0.001
	n	%	n	%	p value	n	%	n	%	p value
Any asthma medication	87/1,456	6.0	23/205	11.2	0.010	94/1,554	6.0	16/107	15.0	0.002
Anti-inflammatory treatment	44/1,456	3.0	16/205	7.8	0.002	51/1,554	3.3	9/107	8.4	0.013

CB-IgE = Cord blood immunoglobulin E;

1 data available for 1195 individuals;

2 data available for 1185 individuals.

The odds ratios for symptoms and medication in relation to *high* levels of CB-IgE and a *positive* family history were relatively unchanged after logistic regression adjusted for potential confounders (Table 4). No interaction was observed between CB-IgE and family history.

**Table 4:** Odds ratios (ORs) with 95% confidence intervals (95% CI) for the risk of respiratory symptoms and the risk of asthma medication (≥2 prescriptions/2006–2008) at the age of 32–34 years in relation to high CB-IgE and a positive family history of asthma.

		CB IgE ≥0.9 kU/l		Family history of asthma	
	Prevalence	Crude OR	Adjusted OR	Crude OR	Adjusted OR
		(95% CI)	(95% CI)	(95% CI)	(95% CI)
Respiratory symptoms due to contact with pollen	179/1,195 (15.0%)	2.01 (1.33–3.05)	2.09 (1.35–3.24)^1^	2.38 (1.38–4.10)	2.28 (1.29–4.02)^1^
Respiratory symptoms due to contact with furred animals	115/1,185 (9.7%)	2.03 (1.25–3.31)	1.99 (1.19–3.33)^1^	3.04 (1.68–5.51)	2.93 (1.58–5.46)^1^
Any asthma medication	110/1,661 (6.6%)	1.98 (1.22–3.21)	1.95 (1.19–3.20)^2^	2.75 (1.55–4.87)	2.67 (1.49–4.78)^2^
Anti-inflammatory treatment	60/1,661 (3.6%)	2.70 (1.50–4.89)	2.64 (1.45–4.83)^2^	2.72 (1.30–5.70)	2.59 (1.20–5.42)^2^

CB-IgE = Cord blood immunoglobulin E.

1 Final model adjusted for gender, having lived on a farm during the first 5 years of life, season of birth, maternal age, Small for Gestational Age, Large for Gestational Age, gestational age, mother’s country of birth, Caesarean section, parity, elevated CB-IgE (≥0.9 kU/l ) or positive family history of asthma.

2 Final model adjusted for gender, season of birth, maternal age, Small for Gestational Age, Large for Gestational Age, gestational age, mother’s country of birth, Caesarean section, parity, elevated CB-IgE (≥0.9 kU/l ) or positive family history of asthma.

The predictive values of elevated CB-IgE or a family history of asthma were low for allergen-induced respiratory symptoms and asthma medication at 32–34 years of age (Table 5). Only 8% of the individuals with elevated cord blood IgE or a family history of asthma in infancy could be linked to current dispensation of anti-inflammatory asthma medication at follow-up.

**Table 5:** Sensitivity, specificity and predictive values of elevated CB-IgE (≥0.9 kU/l) or positive family history of asthma (FHA+) for respiratory symptoms due to pollen or furred pets and dispensed asthma medication (≥2 dispensed prescriptions/3 years) at the ages of 32–34 years.

	CB-IgE+				FHA+			
	SE	SP	PPV	LR+	SE	SP	PPV	LR+
Respiratory symptoms due to pollen	20	89	24	1.8	11	95	28	2.2
Respiratory symptoms due to furred pets	21	89	16	1.8	14	95	23	2.8
Any asthma medication	21	94	11	1.9	15	94	15	5
Anti-inflammatory medication (all cases, n = 60)	27	88	8	2.2	15	94	8	2.5
Anti-inflammatory medication (only new cases; n = 49)^1^	22	88	5	1.9	12	94	6	2.0

CB-IgE = Cord blood immunoglobulin E.

CB-IgE+ = Cb-IgE ≥0.9 kU/l; SE = sensitivity; SP = specificity; PPV = positive predictive value; LR+ = positive likelihood ratio.

1 No asthma reported at previous follow-ups in childhood.

The combination (“and/or”) of these two presumptive factors did not increase the predictive values substantially. If both factors were combined (“and”) the sensitivity (SE) for anti-inflammatory medication was 2, specificity (SP) 99 and positive predictive value (PPV+) 7. If either factor was positive (“or”), the corresponding numbers were SE 40, SP 83 and PPV+8 (data not shown).

Forty-nine of 60 individuals with dispensed anti-inflammatory asthma medication at 32–34 years of age had not been reported having asthma at previous check-ups of the cohort during childhood. Only 5% with elevated cord blood IgE and 6% with a family history of asthma in infancy could be linked to current dispensation of anti-inflammatory asthma medication as adults.

**Figure 2:**
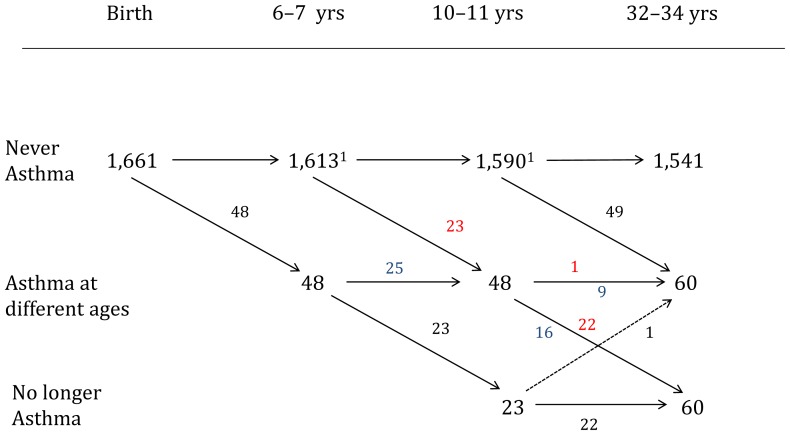
Asthma diagnosis and asthma medication over time. Flow chart of the assessment of asthma status at different ages (asthma diagnosis at 6–7 and 10–11 years and anti-inflammtory treatment at 32–34 years of age). The changes in asthma status of different individuals between the different time points is shown in numbers. Corresponding individuals are marked with **blue** or **red** numbers describing individual changes in asthma status over time. ^1^figures based on the total number of individuals linked to the Swedish Medical Birth Register (n = 1,661).

Figure 2 shows the flow chart of different individuals with asthma diagnosis at 6–7 and 10–11 years of age as well as those who dispensed anti-inflammatory asthma medication at the age of 32–34 years. Most of the cases with dispensed anti-inflammatory asthma medication at 32–34 years of age had not been reported having asthma at 6–7 or 10–11 years of age (49/60). Only nine cases had been diagnosed with asthma on both follow-up occasions in childhood and had been dispensed asthma medication at 32–34 years of age. The proportion of individuals with elevated CB-IgE was somewhat higher among those who had asthma at 6–7 years of age (35%; 17/48) and 10–11 years of age (44%; 21/48) compared to those being dispensed asthma medication at the age of 32–34 years (27%; 16/60). The predictive values for elevated CB-IgE and a positive family history of asthma did not differ significantly when focusing on the 49 new cases (Table 5).

Of the 60 adults with dispensed anti-inflammatory asthma medication at 32–34 years of age, 31 were males and 29 females. Twenty-five of the 49 new cases were males and 24 females. (Table 2). The group of 60 individuals who had an asthma diagnosis at the follow-ups in childhood but had not been dispensed any anti-inflammatory treatment as adults (Figure 2) showed a slight preponderance of males, with 34 men compared with 26 women.

We found an association between a family history of asthma in infancy and anti-inflammatory asthma treatment in adults for men (aOR 4.77) but not for women (aOR 1.13). We did not observe significant gender differences in the association between elevated CB-IgE and anti-inflammatory asthma treatment among adults (Table S2).

## Discussion

In this follow-up at 32–34 years, we were able to link almost all individuals in the original birth cohort to the Swedish Medical Birth Register. Moreover, 72% also responded to a questionnaire on current symptoms. Elevated CB-IgE and a family history of asthma in infancy were associated with a two- to threefold increased risk of dispensed asthma medication and allergy-related respiratory symptoms. However, CB-IgE or reported heredity in infancy was not a good predictor of medication or symptoms in adulthood. Only 21% of all individuals with elevated CB-IgE and 15% with a family history used asthma medication after three decades. Most of the individuals with dispensed anti-inflammatory asthma medication at 32–34 years of age had not been reported having asthma at previous check-ups of the cohort during childhood. To our knowledge, no other birth cohort has been able to elucidate the association between family history of asthma, CB-IgE and asthma disease in adults aged more than 30 years.

Our findings in this adult population are consistent with the results of similar investigations in paediatric populations [1], [4], [10]. Only two studies have investigated the association between CB-IgE, and asthma and allergic disease in adulthood and not beyond the age of 20 years, but their results were contradictory and based on smaller birth cohorts than ours [3], [5]. Pesonen et al. demonstrated that CB-IgE above 0.5 kU/l was associated with a two- to threefold increased risk of allergic symptoms [3], whereas Shah et al. failed to find any association between continuous levels of CB-IgE and different clinical outcomes [5].

Fixed versus continuous cut-off levels of CB-IgE could affect the outcome as could different cut-off levels. In our study the detection level of 0.9 kU/l was given by the calibration of the original laboratory test. As we no longer have access to the blood samples, we were unable to investigate the outcome of continuous levels or a lower cut-off more similar to the one used in several recent studies [1], [3], [4], [10]. A cut-off at 0.9 kU/l means that our study has higher specificity but lower sensitivity than studies based on a cut-off at 0.5 kU/l.

High CB-IgE values could be the result of maternofetal contamination. A Danish cohort study of 200 children with maternal asthma suggested that maternal IgE antibodies contributed to high CB-IgE values in about 50% of all individuals with CB-IgE values over 0.5 kU/l. The suspected contamination was based on the detection of IgA and allergen-induced specific IgE in the sampled cord blood [13]. In contrary, other studies have suggested that maternofetal contamination of cord blood is uncommon. IgA antibodies were detected in less than 5%, even in mother-child pairs with high IgE levels against the same allergen [14]. Low concordance between specific IgE in cord blood and maternal blood for inhalant allergens was demonstrated as an indicator of no significant maternofetal contamination. The agreement between specific IgE for food allergens in cord blood and maternal blood was much better, which the authors interpreted as an indication of sensitization already in utero, especially against food allergens. The high CB-IgE values were considered to be chiefly the result of synthesization by the fetus and not due to maternofetal contamination [14], [15].

It is not easy to elucidate why these different studies have shown such opposing results. However, they differ in several parameters, such as laboratory methods for detecting the different immunoglobulins, the method of securing adequate cord blood samples from the neonates, and the characteristics of the cohorts, just to mention some of the differences between the studies that might have influenced the different results.

As we did not have access to the original blood samples anymore, as mentioned above, we were unable to control for the proportion of IgA in cord blood, which is more or less standard procedure nowadays when investigating the role of CB-IgE. To account for the risk of maternofetal contamination in our study, we excluded subjects with rather high CB-IgE levels (≥10.0 kU/l) as suggested in a previous study [10]. Furthermore, good agreement between high CB-IgE levels and high total IgE levels at the age of 18–24 months was reported in our birth cohort [6] indicating that the majority of high IgE levels in cord blood are of fetal origin. This is in accordance with the findings in the previously mentioned Danish study that found maternofetal contamination in up to 50% of the samples. Bønnelykke et al. found that children with elevated CB-IgE levels and indications of maternofetal contamination had significantly lower IgE levels at 6 months of age compared with children with elevated CB-IgE levels without indications of maternofetal contamination. Furthermore, elevated CB-IgE levels were not significantly associated with IgE levels at 6 months of age in samples with indications of maternofetal transfer of IgE after adjustment for maternal IgE levels, which was in contrast to a highly significant independent association for samples in which transfer was not indicated [13].

Dispensed asthma medication was less common than reported allergy-related respiratory symptoms, and the two outcomes only partly overlapped. The prevalence rates of respiratory symptoms were 10–15%, and only 20–30% of those with reported symptoms had purchased asthma medication. Our definition of allergy-related respiratory symptoms included wheezing, breathlessness or coughing. Some individuals might have reported non-asthmatic symptoms from the airways, and others had mild symptoms with no need of medication. In contrast, allergy-related respiratory symptoms were reported by 50–70% of those who had purchased *any asthma medication*. This is in line with reviews indicating that only approximately 50% of all asthma is associated with allergy [16]. The prevalence of *any asthma medication* use was fairly similar to the prevalence rates of physician-diagnosed asthma in recent studies among young adults in Sweden [17], [18].

Access to data from the Swedish Medical Birth Register in this study gave us the opportunity to control for numerous potential confounding factors that directly or indirectly could have influenced the outcome. Adjusting for these factors, however, altered the odds ratios only slightly. Elevated CB-IgE and the family history based on information collected in early life were highly significant and independent risk factors for asthma medication and respiratory symptoms in adulthood. The odds ratios for both outcomes were fairly similar even though they were markers of partly different entities. We also investigated whether elevated CB-IgE and a family history of asthma based on information collected in early life could be used for screening. However, and in line with previous studies in younger adults [3], [5], neither elevated CB-IgE nor family history were efficient enough to identify individuals with asthma in adulthood. That did not change either when focusing on the cases with asthma medication at 32–34 years of age who had not been reported to have asthma in childhood, probably as the majority of adult cases with dispensed asthma medication are new cases (Figure 2). We noticed also a reduced proportion of subjects with elevated CB-IgE among those with dispensed asthma medication as adults compared to the subjects with an asthma diagnosis in childhood, which explains the poor predictive values of these parameters. The predictive ability did not improve even when family history and CB-IgE were combined. Sensitivity, however, did increase to a certain extent when elevated CB-IgE *or* a positive family history of asthma were used as a combined information with a sensitivity of 40 for anti-inflammatory medication and only a marginally reduced specificity of 83. Still, the positive predictive value did not increase. In our population-based birth cohort with a prevalence of asthma around 12.0% our investigated parameters do not predict the value of interest in a sufficient way, but might be useful in subgroups with higher asthma prevalence as e.g. high-risk infants.

Further limitations of our study need to be commented on. We have used questionnaire-reported symptoms induced by furred pets or pollen as markers of allergic symptoms from the airways. Other potential allergens as for example molds and house dust mites were not included. House dust mites, however, are no common cause of asthma in this part of Europe. The questionnaire lacked specific questions about physician-diagnosed asthma, which is a weakness we cannot fully compensate for, and which might have contributed to non-overlap between allergen-induced asthma symptoms recorded in the questionnaires and asthma medication data from the drug register. Prescription data probably reflect an asthma diagnosis better than self-reported respiratory symptoms, which more often include other respiratory symptoms than asthma. Furthermore, the design of the follow-up did not include more objective measurements of sensitization such as skin prick tests or specific IgE values. Sensitization to common aeroallergens is a known risk factor for the development of asthma [19]. However, atopy is not present in all cases of asthma and the problems of misclassification are obvious [20], [21], [22].

As misclassification of our outcome variable may reduce the estimated effect, we also investigated the dispensation of anti-asthmatic medication as a proxy for asthma disease similar to other register-based studies investigating asthma in adolescents and young adults [23], [24], [25]. Even this outcome variable has certain limitations, mainly related to potential over- or undertreatment of respiratory symptoms [26]. However, this seems to be a minor problem in the present study as rather good agreement has been demonstrated between register-based data on dispensed prescriptions of anti-asthmatic drugs and physician-diagnosed asthmatic disease [27]. Further, reported anti-asthmatic drug use has been found to be an accurate indicator of both actual use of medication and asthma disease [28]. In Sweden, a prescription is valid for one year, with one filling usually covering a 3-month consumption period. Occasionally, however, a physician may prescribe inhaled steroids to be dispensed once only to test non-specific respiratory symptoms, although this is rather unusual in adult patients, who are also unlikely to purchase a drug more than once if it failed to help the first time. We have used at least two purchases of prescribed asthma medication as a proxy for asthma to avoid even this type of bias. The use of so-called objective measurements, such as bronchial hyper-responsiveness, can be questioned, at least in an epidemiological setting, because they do not necessarily increase diagnostic accuracy [29]. As there is currently no “golden standard” for the definition of asthma in epidemiological studies, different measurements can and should be used as long as the investigator is aware of each measurement’s particular weaknesses. As prescription data are collected prospectively and independently from the studied individuals, they represent unbiased data. We believe, therefore, that our outcome variable of dispensed asthma medication probably represents current disease more accurately than self-reported data, especially in the case of anti-inflammatory treatment. It is predominantly prescribed to patients with active disease [24] and is more likely to be an indicator of clinically significant disease [30].

Information about asthma heredity was based on data collected by parental questionnaires when the individuals were about 18 months of age. The parents simply answered the question if they, their own parents or any of the child’s siblings ever had asthma. Age and sex of the siblings or numbers of siblings in the family were not recorded which might limit the significance of our variable. The aim of our analysis, however, was to assess if data about asthma disease in the family which can be easily and quickly collected by a physician when meeting a child and its parents could have any diagnostic value for the future.

Adult-onset asthma is known to be more prevalent in females than in males, in contrast to asthma before puberty. The reasons for this are multiple and include sex hormone influences, smoking habits, genetic and immunological factors, among others [31], [32]. We found no clear gender difference among those individuals with asthma as adults, neither in the whole group (n = 60) nor in the sub-group of 49 individuals developing asthma after the follow-ups in childhood (Figure 2). The latter group might include individuals who developed asthma even before puberty but who could not be recognized as such due to the lack of a follow-up between the age of 10–11 years and adulthood.

In the group of individuals who no longer have asthma but had asthma at the time of follow-up in childhood, a slight predominance of males over females was seen (34 versus 26). This supports previous discussions about girls having persistent asthma beyond puberty whereas boys more often show a decrease in symptoms with increasing age [32], [33].

Gender-specific analyses in our study revealed an association between a family history of asthma and anti-inflammatory asthma treatment in adults for men but not for women. The reason for this difference is not obvious. It has been suggested that women have an increased risk of developing non-allergic asthma, which is why the phenotype of asthma in our adult population could influence our findings [34]. The nature of our outcome variable, however, does not allow a distinction between non-allergic and allergic asthma. The association between elevated CB-IgE and anti-inflammatory asthma treatment among adults did not to differ greatly between different sexes (Table S2).

### Conclusions

Elevated CB-IgE and a family history of asthma at birth were associated with dispensed asthma medication and questionnaire-reported allergen-induced respiratory symptoms in adulthood. However, neither family history nor CB-IgE was useful for screening as their predictive ability in this population-based sample was poor.

## Supporting Information

Table S1 **Individuals with a positive family history of asthma at the age of 18 months ( = at least one parent or sibling with asthma reported).** QN = questionnaire.
(DOC)

Table S2 **Gender specific odds ratios (ORs) with 95% confidence intervals (95% CI) for the risk of dispensed anti-inflammatory asthma medication (≥2 prescriptions/2006–2008) at the age of 32–34 years in relation to high CB-IgE and a positive family history of asthma.** CB-IgE = Cord blood immunoglobulin E 1Final model adjusted for season of birth, maternal age, Small for Gestational Age, Large for Gestational Age, gestational age, mother’s country of birth, Caesarean section, parity, elevated CB-IgE (≥0.9 kU/l) or a positive family history of asthma.
(DOC)
