# Supplementary material for: Asthma Heredity, Cord Blood IgE and Asthma-Related Symptoms and Medication in Adulthood: A Long-Term Follow-Up in a Swedish Birth Cohort
Source: PLoS One. 2013 Jun 21;8(6):e66777. doi: 10.1371/journal.pone.0066777 (PMC3689672; doi:10.1371/journal.pone.0066777)
Supplement: Table S1 — Individuals with a positive family history of asthma at the age of 18 months ( = at least one parent or sibling with asthma reported). QN = questionnaire. (DOC) [file pone.0066777.s001.doc]

| Table S1: Individuals with a positive family history of asthma at the age of 18 months (= at least one parent or sibling with asthma reported) | | | | | | | |
| --- | --- | --- | --- | --- | --- | --- | --- |
|  | QN + register-linked | Register-linked | Maternal asthma |  | Paternal asthma |  | Sibling with asthma |
|  | n = 1,227 | n = 1,661 |  |  |  |  |  |
|  |  |  |  |  |  |  |  |
|  | 2 | 3 | X |  |  |  | X |
|  | 2 | 2 |  |  | X |  | X |
|  | 20 | 26 |  |  |  |  | X |
|  | 24 | 39 | X |  |  |  |  |
|  | 24 | 35 |  |  | X |  |  |
|  | 2 | 2 | X |  | X |  |  |
|  |  |  |  |  |  |  |  |
| Total | 74 (6.0%) | 107 (6.4%) |  |  |  |  |  |
| QN = questionnaire | | | | | | | |
