# Supplementary material for: Asthma Heredity, Cord Blood IgE and Asthma-Related Symptoms and Medication in Adulthood: A Long-Term Follow-Up in a Swedish Birth Cohort
Source: PLoS One. 2013 Jun 21;8(6):e66777. doi: 10.1371/journal.pone.0066777 (PMC3689672; doi:10.1371/journal.pone.0066777)
Supplement: Table S2 — Gender specific odds ratios (ORs) with 95% confidence intervals (95% CI) for the risk of dispensed anti-inflammatory asthma medication (≥2 prescriptions/2006–2008) at the age of 32–34 years in relation to high CB-IgE and a positive family history of asthma. CB-IgE = Cord blood immunoglobulin E 1Final model adjusted for season of birth, maternal age, Small for Gestational Age, Large for Gestational Age, gestational age, mother’s country of birth, Caesarean section, parity, elevated CB-IgE (≥0.9 kU/l) or a positive family history of asthma. (DOC) [file pone.0066777.s002.doc]

| TABLE S2: Gender specific odds ratios (ORs) with 95% confidence intervals (95% CI) for the risk of dispensed anti-inflammatory asthma medication (≥2 prescriptions/2006–2008) at the age of 32–34 years in relation to high CB-IgE and a positive family history of asthma. | | | | | |
| --- | --- | --- | --- | --- | --- |
|  |  | CB IgE ≥ 0.9 kU/l | | Family history of asthma | |
|  | Prevalence | Crude OR | Adjusted OR | Crude OR | Adjusted OR |
|  |  | (95% CI) | (95% CI) | (95% CI) | (95% CI) |
|  |  |  |  |  |  |
| Males | 31/1,661  (1.9%) | 2.46 (1.10-5.47) | 2.31 (1.00-5.34)^1^ | 4.32 (1.78–10.50) | 4.77 (1.87–12.21)^1^ |
|  |  |  |  |  |  |
| Females | 29/1,661 (1.7%) | 3.10 (1.28–7.50) | 3.10 (1.25–7.71)^1^ | 1.18 (0.27–5.11) | 1.13 (0.25–5.16)^1^ |
|  |  |  |  |  |  |
| CB-IgE = Cord blood immunoglobulin E | | | | | |
| ^1^Final model adjusted for season of birth, maternal age, Small for Gestational Age, Large for Gestational Age, gestational age, mother’s country of birth, Caesarean section, parity, elevated CB-IgE (≥ 0.9 kU/l) or a positive family history of asthma | | | | | |
